# Green cocoon-derived sericin reduces cellular damage caused by radiation in human keratinocytes

**DOI:** 10.1038/s41598-024-53712-x

**Published:** 2024-02-06

**Authors:** Nahoko Kakihara, Momoko Sato, Ayaki Shirai, Mizuki Koguchi, Shiori Yamauchi, Toshimichi Nakano, Ryuta Sasamoto, Hideyo Sato

**Affiliations:** 1 Department of Nursing, Graduate School of Health Sciences, Niigata University, Niigata, Japan; 2 Department of Medical Technology, Faculty of Medicine, Niigata University, Niigata, Japan; 3 Department of Radiology and Radiation Oncology, Niigata University Graduate School of Medical and Dental Sciences, Niigata, Japan; 4 Department of Radiological Technology, Graduate School of Health Sciences, Niigata University, Niigata, Japan

**Keywords:** Health care, Materials science

## Abstract

Radiation therapy used in the treatment of cancer causes skin damage, and no method of care has been established thus far. Recently, it has become clear that sericin derived from silkworm cocoons has moisturizing and antioxidant functions. In addition, green cocoon-derived sericin, which is rich in flavonoids, may have enhanced functions. However, whether this green cocoon-derived sericin can reduce radiotherapy-induced skin damage is unclear. In the present study, we aimed at establishing care methods to reduce skin cell damage caused by X-irradiation using green cocoon-derived sericin. We investigated its effect on human keratinocytes using lactate dehydrogenase activity to indicate damage reduction. Our results showed that green cocoon-derived sericin reduced cell damage caused by X-irradiation. However, this effect was not observed when cells were treated before X-irradiation or with a sericin derived from white cocoons. In addition, green cocoon-derived sericin decreased the levels of reactive oxygen species and lipid peroxidation. Our results suggest that green cocoon sericin mitigates the damaging effect of X-irradiation on cells, hence presenting potential usefulness in reducing skin damage from radiation therapy and opening new avenues in the care of cancer patients.

## Introduction

Radiotherapy, along with surgery and anticancer drugs, is one of the most important cancer treatment modalities^1,2^. One disadvantage of radiotherapy is the skin damage provoked in the irradiated area^3^. As no standard care method has been established for skin damaged by radiation, these lesions are classified and treated as dry skin caused by damage to sebaceous glands in the skin. Therefore, application of moisturizers such as heparin analogs, which only treat symptoms, is currently used as a common treatment^4^. Indeed, moisturizers can improve skin dryness and desquamation and reduce pain. However, radiation-induced skin damage differs from normal dry skin in its mechanism. Death of cellular tissue, damage to the antioxidant system, DNA damage, and regression of hair follicles occur in irradiated skin^5^, with acute adverse events occurring from the start of the treatment to around immediately after its completion and late adverse events occurring 3 months to several years after the treatment^6,7^. Destroyed skin tissue does not regenerate until cells are replaced by skin turnover^8^. Plasminogen, a proinflammatory factor, is required for the induction of inflammation after irradiation, and inhibition of plasminogen activation has been found to inhibit and prevent the development of radiation dermatitis^9^. Recently, Gupta et al.^10^ reported that the application of Ayurvedic oil preparations (Jatyadi ghrita and Jatyadi tail) promotes healing by stimulating re-epithelialization and anti-inflammation, inhibiting collagen fiber deposition, and reducing TGF-β1 expression in a rat skin model. Their results suggest that these oil preparations may be useful for human skin injury provoked by radiation.

Silk is a natural composite fiber mainly composed of hydrophobic fibroin and hydrophilic sericin, and produced by the silkworm *Bombyx mori*^11^. Sericin binds and coats the two fibroin filaments in raw silk. Sericin is a high molecular weight granular protein with adhesive and gelatin-like properties, and is highly hydrophilic. Approximately 90% of sericin is composed of hydroxy amino acids (serine and threonine) and polar amino acids. At least three genes are responsible for sericin synthesis, i.e., *Ser1*, *Ser2*, and *Ser3*, and sericin is produced through the alternative splicing of these genes^12^. In the past, sericin was peeled off and discarded during the silk production refinement process; however, recent studies have shown interest in its biological and biomedical applications as it possesses moisturizing, antioxidant, and UV absorbing functions^13–15^. Various cocoon color mutants exist, including white, yellow, golden yellow, orange, pinkish, and green^16^. Cocoon colors are mainly determined by two pigments present in mulberry leaves eaten by silkworms (carotenoids and flavonoids). Notably, flavonoids absent from the mulberry leaf diet have been observed in some silkworm varieties. Among various cocoon colors, green cocoons are rich in flavonoids^10^. Flavonoids are polyphenols with diverse structures that exhibit a wide variety of biological activities, including antioxidant and anti-inflammatory effects^11,12^. Since inflammation is involved in radiation-induced skin damage, we hypothesized that green cocoon-derived sericin, which is rich in flavonoids, may affect such skin damage. To the best of our knowledge, no study has investigated these effects. In the present study, we investigated the possibility that green cocoon-derived sericin (green cocoon sericin) could reduce radiation-induced damage to skin cells and promote healing recovery using human-derived keratinocytes.

## Results

### Green cocoon sericin has protective effects on cellular injury after X-irradiation in human keratinocytes

**Figure 1:**
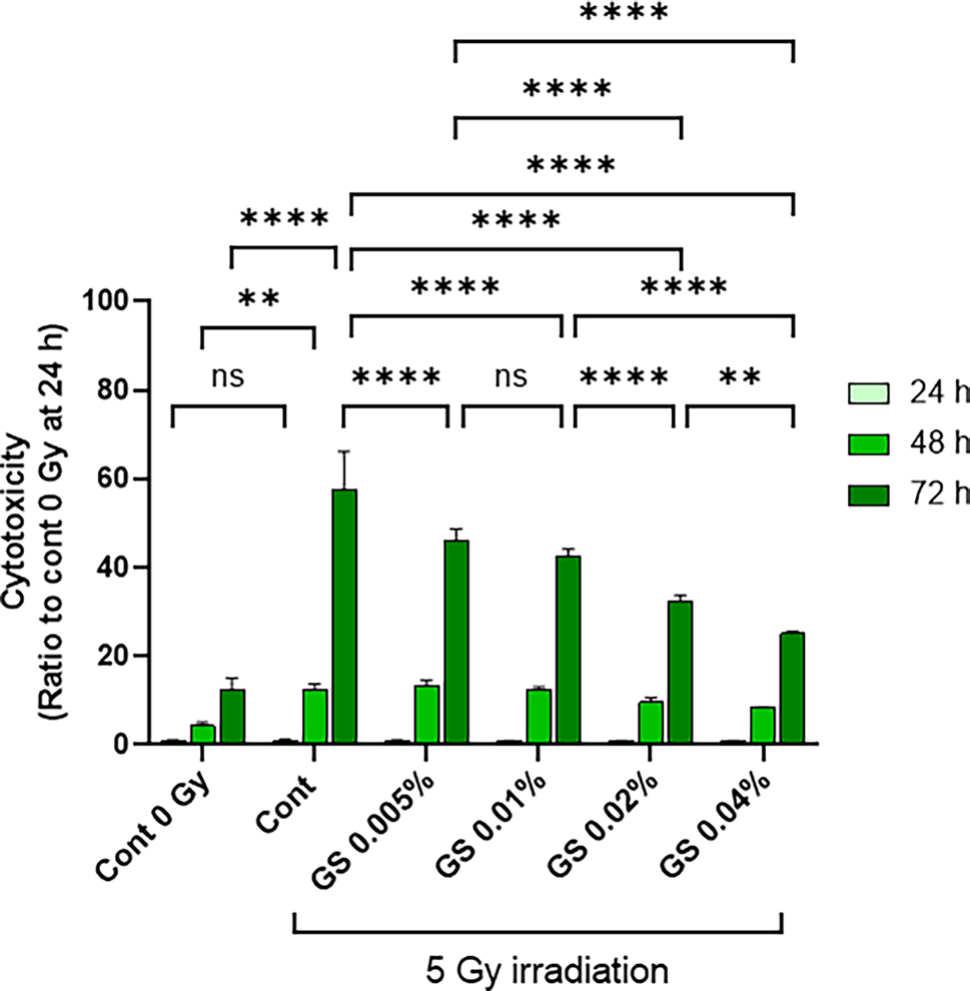
Green cocoon sericin has concentration-dependent protective effects on cellular injury after X-irradiation in human keratinocytes. PSVK1 cells were incubated for 72 h after plating; the cells were then irradiated with 5 Gy. The cells were subsequently cultured with 0.005%, 0.01%, 0.02%, or 0.04% sericin, and LDH in the medium was measured at 24 h, 48 h, and 72 h after X-irradiation. Values are the ratio to LDH activity in the medium of the cells without X-irradiation (Cont 0 Gy), and represented the means ± S.D. of at least three determinations. **p < 0.01, ****p < 0.001, ns indicates no significance.

**Figure 2:**
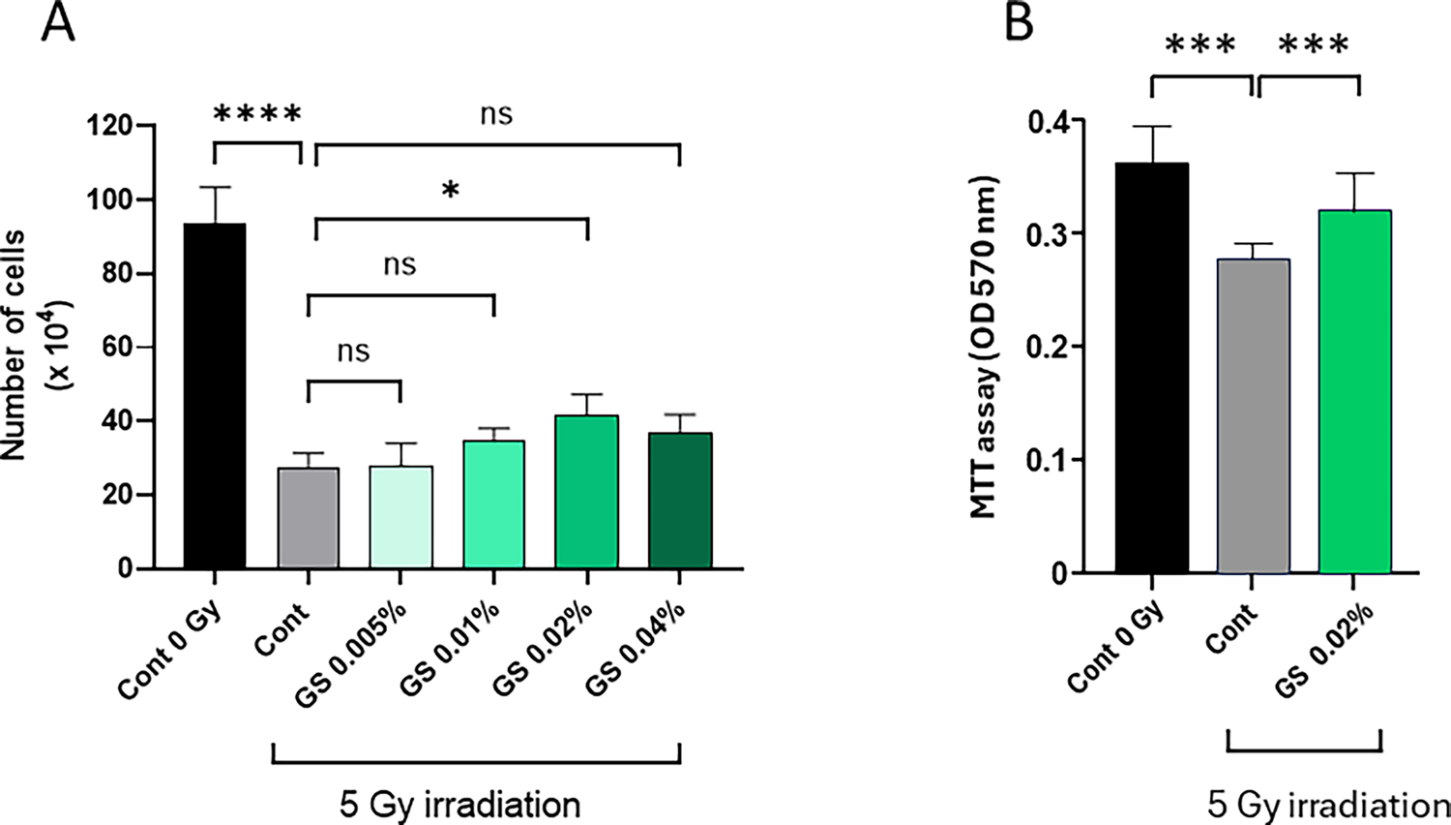
Concentration-dependent cytoprotective effect of green cocoon sericin on cell proliferation. (**A**) After measuring LDH at 72 h in Fig. 1, PSVK1 cells were treated with 0.25% trypsin-EDTA and the detached cells were resuspended in 1 mL of fresh medium, and 50 µL of the suspension was mixed with an equal volume of 0.1% trypan blue solution. Trypan blue-negative cells as viable cells were counted using a hemocytometer. Values show the number of viable cells, and represented the means ± S.D. of at least three determinations. *p < 0.05, ****p < 0.001, ns indicates no significance. (**B**) Cells were irradiated with 5 Gy X-irradiation (or not, Cont 0 Gy), and cultured without (Cont) or with 0.02% sericin (GS 0.02%) for 72 h. Next, cell viability was measured using an MTT assay. Data are represented as mean ± S.D. of at least three technical replicates. ***p < 0.005.

To assess the protective effect of green cocoon sericin against irradiation damage, we measured cell viability through lactate dehydrogenase activity (LDH), a common method used to measure metabolic activity of a tissue. Human keratinocytes (PSVK1 cells) were irradiated with 5 Gy of X-rays. After irradiation, 0.005–0.04% of green cocoon sericin (GS) was immediately added to the medium, and cells were cultured in the presence of GS for 72 h. The culture medium was collected at 24, 48, and 72 h after irradiation, and LDH activity in the culture medium was measured (Fig. 1). No significant difference in LDH activity was observed at 24 and 48 h compared with and control cells (Fig. 1, light green and green, respectively). At 72 h after irradiation, LDH activity clearly decreased with sericin concentration in sericin-treated groups compared with the control group (Fig. 1, dark green; control cytotoxicity versus GS 0.005–0.04%, *p* values < 0.0001, n = 4–8). We counted the number of viable cells at 72 h after irradiation for each GS concentration (Fig. 2A). The number of viable cells tended to increase with GS concentration; we observed a statistically significant increase in the number of cells relative to the control following the addition of 0.02% GS. MTT assay was also performed. Cell viability following X-irradiation was significantly higher in the group with the addition of 0.02% GS than without GS (Fig. 2B). Considering that we could see a statistically significant difference in LDH and also viable cell number between 0.02% sericin added and control, we decided to use 0.02% sericin in subsequent experiments.

### Sericin cytoprotective effect on reducing cellular injury is efficient on different X-rays doses

**Figure 3:**
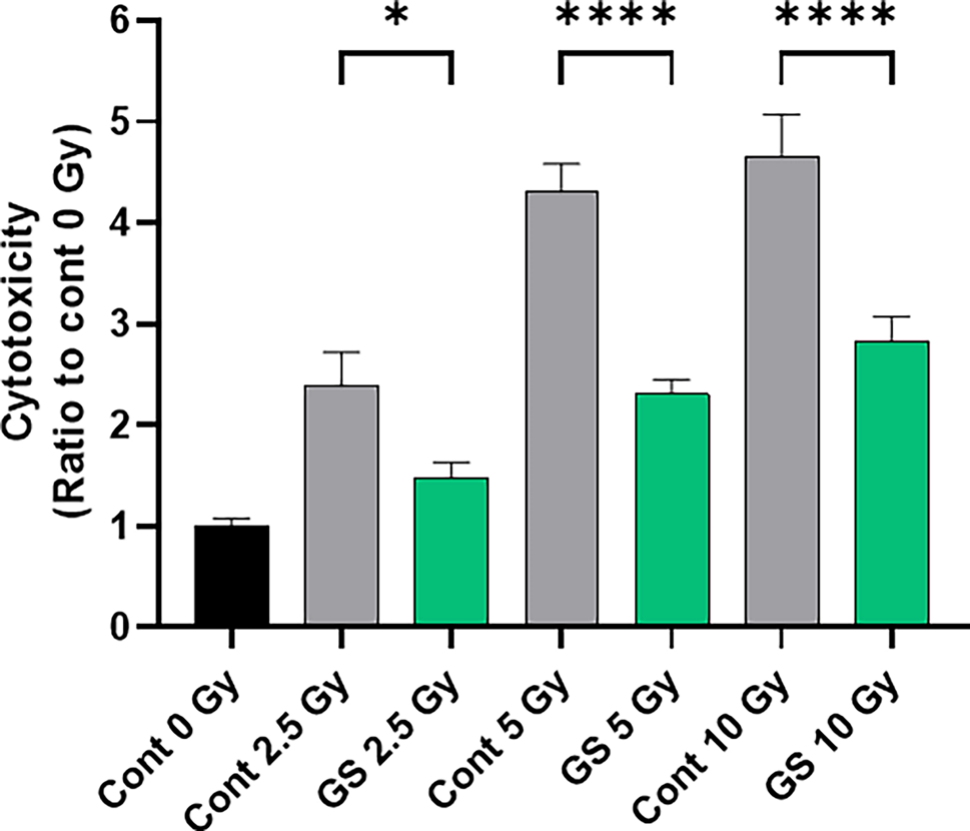
Sericin cytoprotective effect on reducing cellular injury is efficient on different X-ray doses. PSVK1 cells were incubated for 72 h, and then irradiated with 2.5, 5, or 10 Gy. After culturing for another 72 h with 0.02% sericin, LDH in the medium was measured. Values correspond to the ratio of LDH activity in the medium of the different groups compared to the nonirradiated cells n (Cont 0 Gy), and represented the means ± S.D. of at least three determinations. *p < 0.05, ****p < 0.001.

We subsequently aimed to assess the cell protective effects of GS when different doses of X-ray were applied. PSVK1 cells were irradiated with doses between 2.5 and 10 Gy X-rays and incubated without sericin (Fig. 3, gray bars) or with 0.02% sericin (Fig. 3, green bars) for another 72 h. LDH activity was then measured and showed that increased doses of irradiation resulted in increased cytotoxicity, and that the cytoprotective effect of sericin was observed at all doses of X-rays (Fig. 3). These results indicate that the cytoprotective effect of sericin is effective at both low and high irradiation doses.

### Delayed treatment with GS presents an improved efficacy against X-irradiation cytotoxicity

**Figure 4:**
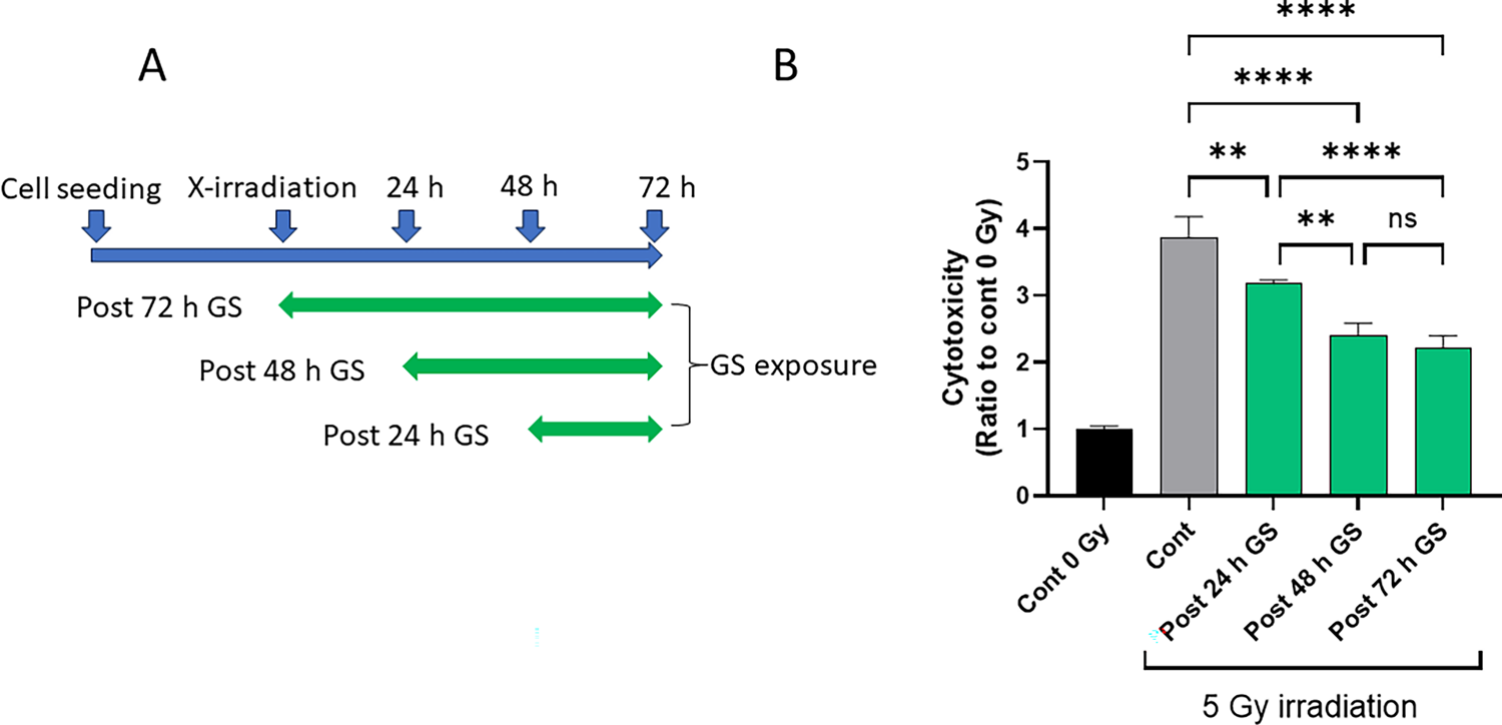
Delayed treatment with GS presents an improved efficacy against X-irradiation cytotoxicity. (**A**) the scheme of the experimental design. PSVK1 cells were incubated for 72 h, and then irradiated with 5 Gy. After the irradiation, cells were further cultured for 72 h without sericin (Cont), for 72 h with 0.02% sericin (post 72 h GS), for further 24 h without sericin and then for 48 h with 0.02% sericin (post 48 h GS), and for further 48 h without sericin and then for 24 h with 0.02% sericin for 24 h (post 24 h GS). (**B**) LDH in the medium was measured. Values are the ratio to LDH activity in the medium of the cells without X-irradiation (Cont 0 Gy), and represented the means ± S.D. of at least three determinations. **p < 0.01, ****p < 0.001, ns indicates no significance.

Next, we aimed to determine the time course of green sericin’s protective effect against X-irradiation. All LDH activity measurements were performed 72 h after irradiation and only the incubation time without (pre-) and with (post-) GS treatment varied. Three groups were prepared considering the incubation time: (1) Post 24 h GS = 48 h pre-GS and 24 h post GS; (2) Post 48 h GS = 24 h pre-GS and 48 h post-GS; (3) Post 72 h GS = 0 h pre-GS and 72 h post-GS (Fig. 4A). The results revealed that the longer the cells were exposed to GS, the higher was the cytotoxicity protective effect (Fig. 4B). However, these results also indicated that this effect was observed even when cells are not exposed directly to GS, and when the delayed exposure period is short.

### GS does not prevent cell damages provoked by X-irradiations when cells are exposed to sericin prior to X-irradiation

**Figure 5:**
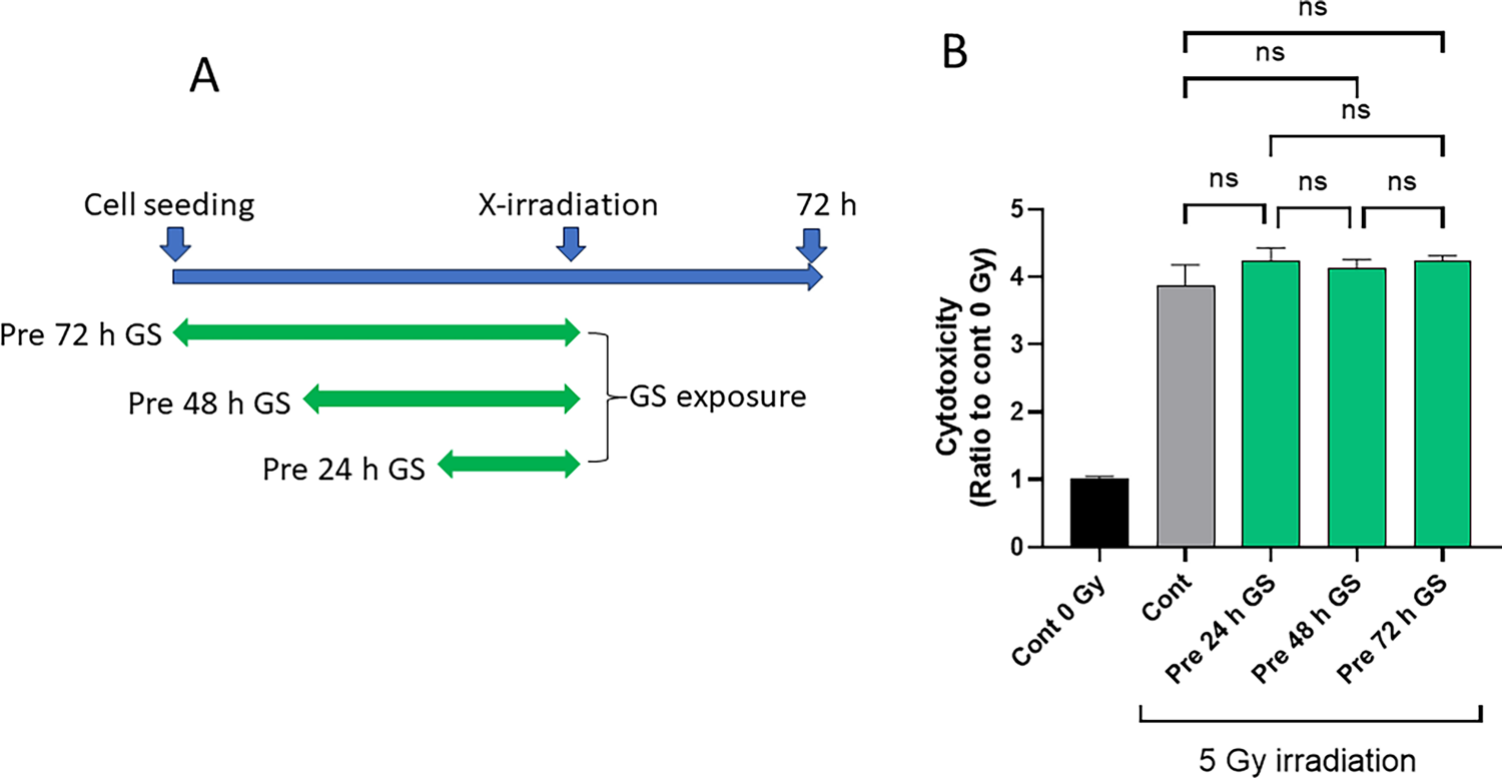
GS does not prevent cell damage provoked by X-irradiation when cells are exposed to sericin beforehand. (**A**) scheme depicting the experimental design. PSVK1 cells were plated and sericin was either added immediately (i.e., Pre 72 h GS), 24 h later (i.e., Pre 48 h GS), or 48 h later (Pre 24 h GS). Cells were then cultured for another 24 h, and then irradiated with 5 Gy. After X-irradiation, cells were cultured for another 72 h without sericin. The LDH levels of the medium were then measured. (**B**) measurements of LDH in the medium. Values show the ratio of LDH activity of the media of irradiated cells to that of cells without X-irradiation (Cont 0 Gy). Data indicate the mean ± S.D. of at least three technical replicates, ns indicates no statistically significant difference.

The cytoprotective effect of GS was also examined when cells were exposed to GS prior to X-irradiation. GS was added immediately after seeding the cells (Fig. 5, Pre 72 h GS), 24 h later (Fig. 5, Pre 48 h GS), or 48 h after seeding (Fig. 5, Pre 24 h GS). All the cells were cultured for another 24 h, and then the medium was replaced to new one (remove GS) and cells were irradiated with X-rays. LDH activity of each group was measured 72 h after X-irradiation. The results showed that pretreatment with GS before X-irradiation had no cytoprotective effect.

### Cytoprotective effect of white cocoon-derived sericin in cellular injury by X-irradiation

**Figure 6:**
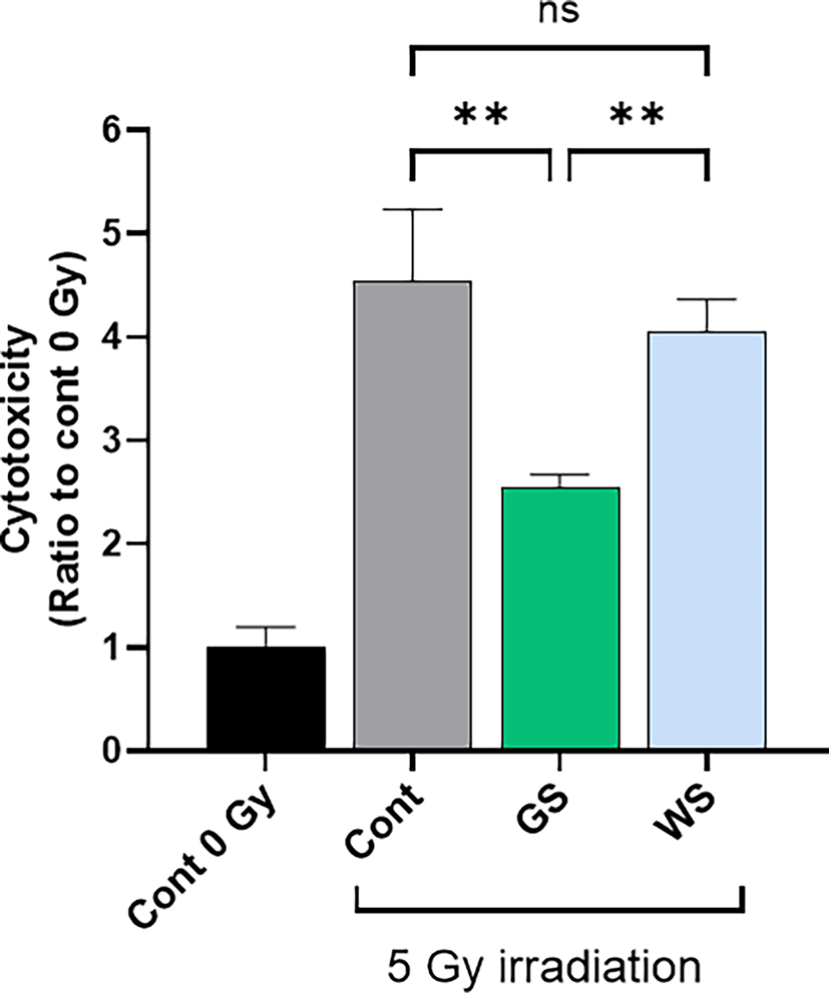
Cytoprotective effect of white cocoon sericin in cellular injury by X-irradiation. PSVK1 cells were incubated for 72 h, and then irradiated with 5 Gy. After culturing further 72 h without sericin (Cont), with 0.02% green cocoon sericin (GS), or 0.02% white cocoon sericin (WS), LDH in the medium was measured. Values are the ratio to LDH activity in the medium of the cells without X-irradiation (Cont 0 Gy), and represented the means ± S.D. of at least three determinations. **p < 0.01, ns indicates no significance.

Currently, the main cocoons of silkworms kept in the sericulture industry are white. White cocoon-derived sericin (WS) shows moisturizing effect, antioxidant character, and mitogenic effect on mammalian cells^17^. To assess whether WS also presents irradiation damage protective effect, we compared LDH activity in X-irradiated cells treated with either GS or WS. As shown in Fig. 6, no significant cytoprotective effect was observed in cells treated with WS compared with control and GS. GS is enriched in flavonoids^18^. Therefore, these results may be attributable to flavonoids in the green sericin fraction.

### Cytoprotective effect of dialyzed green cocoon sericin against cellular injury caused by X-irradiation

**Figure 7:**
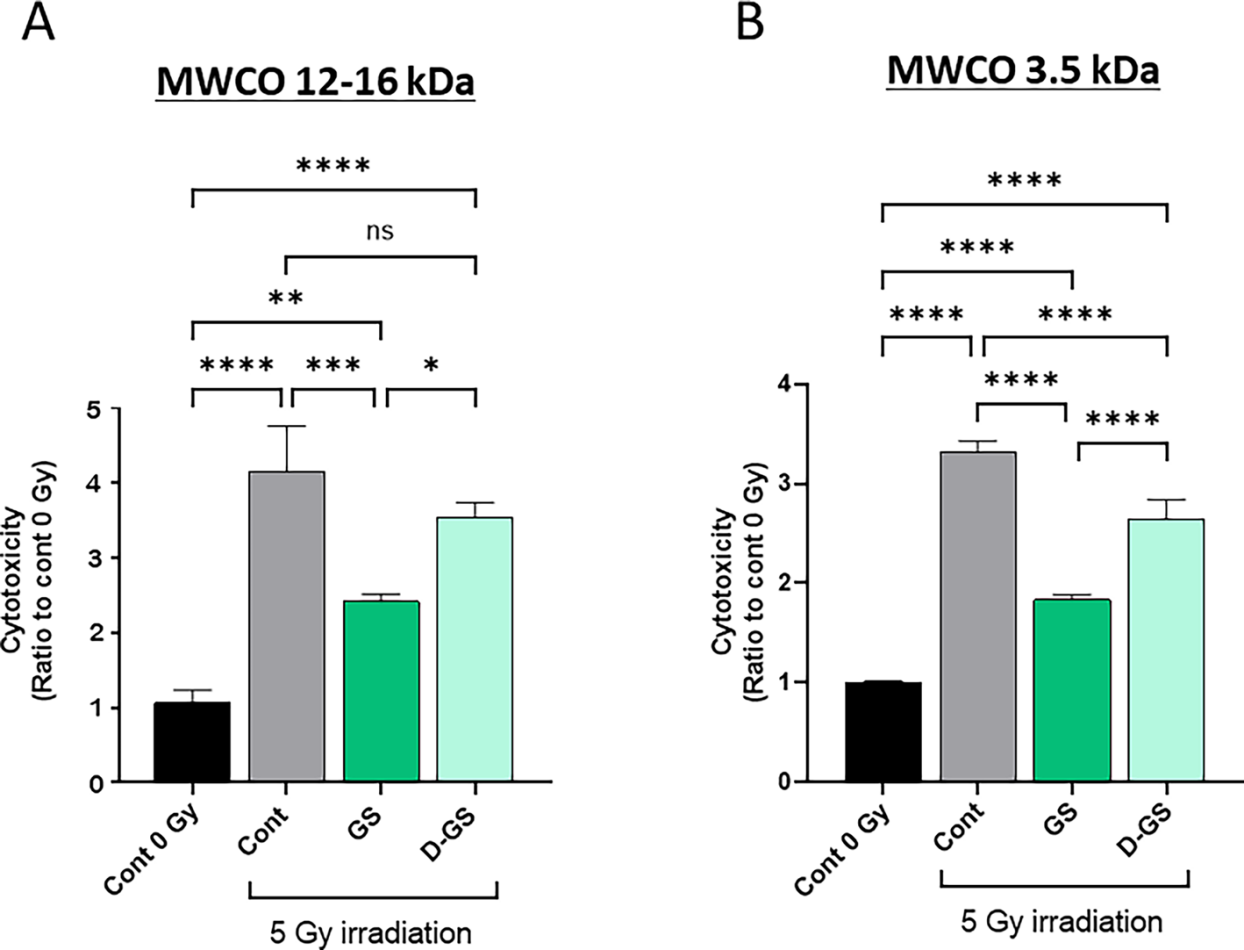
Cytoprotective effect of dialyzed green cocoon sericin in cellular injury by X-irradiation. Cytoprotective effect of green cocoon sericin after dialyzing with a dialysis membrane with MWCO 12,000–16,000 (A) or with a dialysis membrane with MWCO 3500 (B). PSVK1 cells were incubated for 72 h after plating, and then the cells were irradiated with 5 Gy. After culturing further 72 h without sericin (Cont), with 0.02% green cocoon sericin (GS), or 0.02% dialyzed green cocoon sericin (D-GS), LDH in the medium was measured. Values are the ratio to LDH activity in the medium of the cells without X-irradiation (Cont 0 Gy), and represented the means ± S.D. of at least three determinations. *p < 0.05, **p < 0.01, ***p < 0.005, ****p < 0.001, ns indicates no significance.

**Figure 8:**
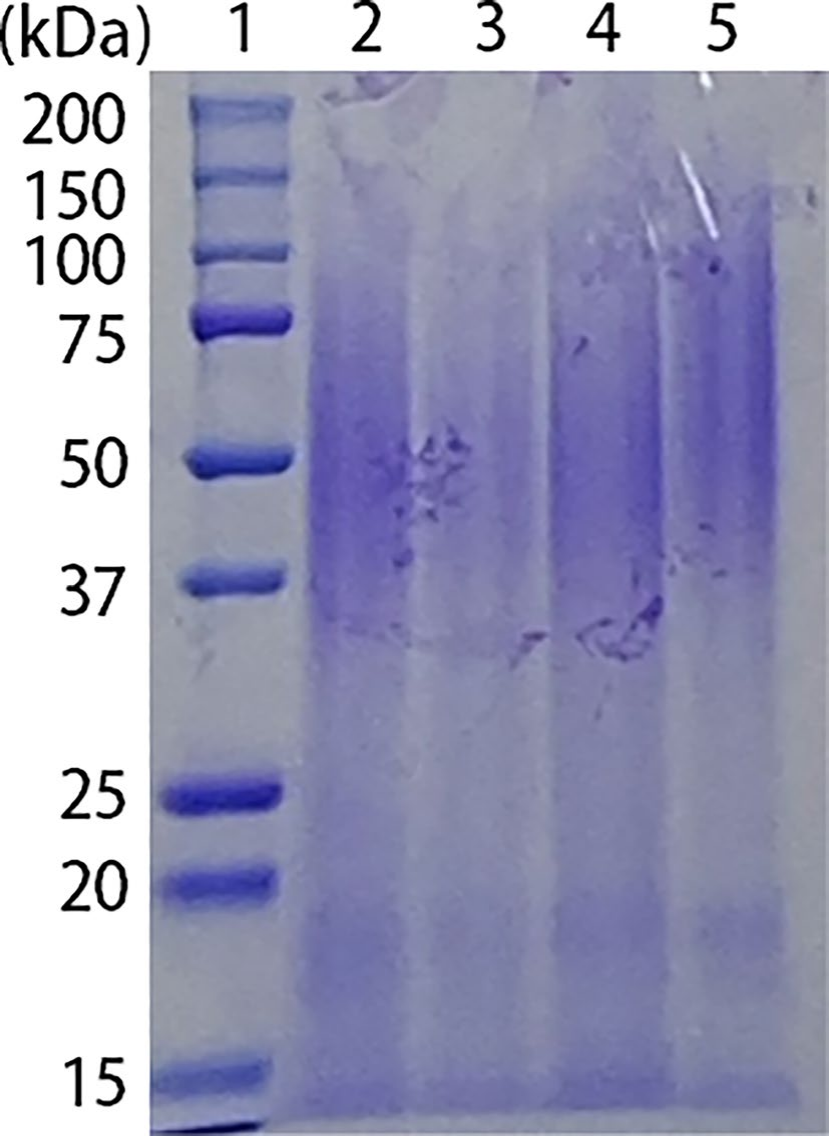
Electrophoresis of dialyzed and nondialyzed sericin. Untreated green cocoon sericin was dialyzed using a membrane with MWCO 12,000–16,000, or with MWCO 3500. Green and white cocoon sericin samples were first mixed with a protein denaturing solution and electrophoresed; subsequently, the gel was stained with Coomassie Brilliant Blue. Samples were as follows: Lane 1; molecular weight marker, lane 2; nondialyzed green cocoon sericin, lane 3; green cocoon sericin dialyzed with MWCO 3500 membrane, lane 4; green cocoon sericin dialyzed with MWCO 12,000–16,000 membrane, lane 5; white cocoon sericin.

To investigate what might be responsible for the cytoprotective effect of GS against X-irradiation, the cytoprotective effect of GS dialysis product was investigated. GS was dialyzed overnight on different molecular weight cutoffs (MWCO) dialysis membranes. The results showed that the cytoprotective of dialyzed GS effect was almost lost when using a dialysis membrane with MWCO 12–16 kDa (Fig. 7A). On the contrary, when using a dialysis membrane with MWCO 3500, the resulting dialyzed GS product presented a significant cytoprotective effect compared with control (5 Gy without sericin). However, this cytoprotective effect was reduced compared to not-dialyzed GS (Fig. 7B). We examined whether dialysis of GS caused differences in electrophoretic patterns. No difference was found in the sericin portion when dialyzed on either membrane (Fig. 8). The electrophoretic pattern of WS was also not significantly different from that of GS. These findings suggest that the components of GS responsible for the cytoprotective effect have a molecular weight below 3500 and between 3500 and 12,000.

### Reduction of reactive oxygen species levels and lipid peroxidation by green cocoon sericin

**Figure 9:**
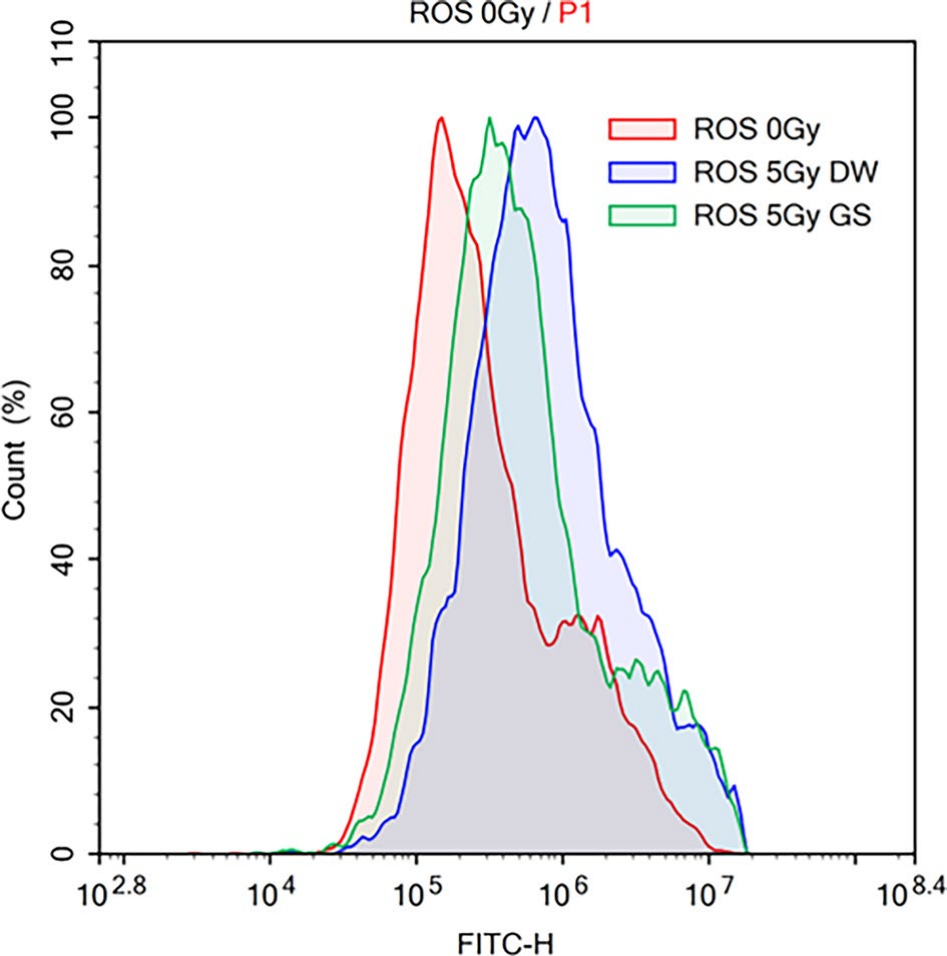
Assessments of intracellular reactive oxygen species. After X-irradiation at 5 Gy, cells were cultured for 72 h with or without 0.02% green cocoon sericin. We then quantified the intracellular reactive oxygen species (ROS) levels in each sample. Data represent ROS levels in non-X-irradiated cells (red), in X-irradiated cells without sericin (blue), and in X-irradiated cells with sericin (green).

**Figure 10:**
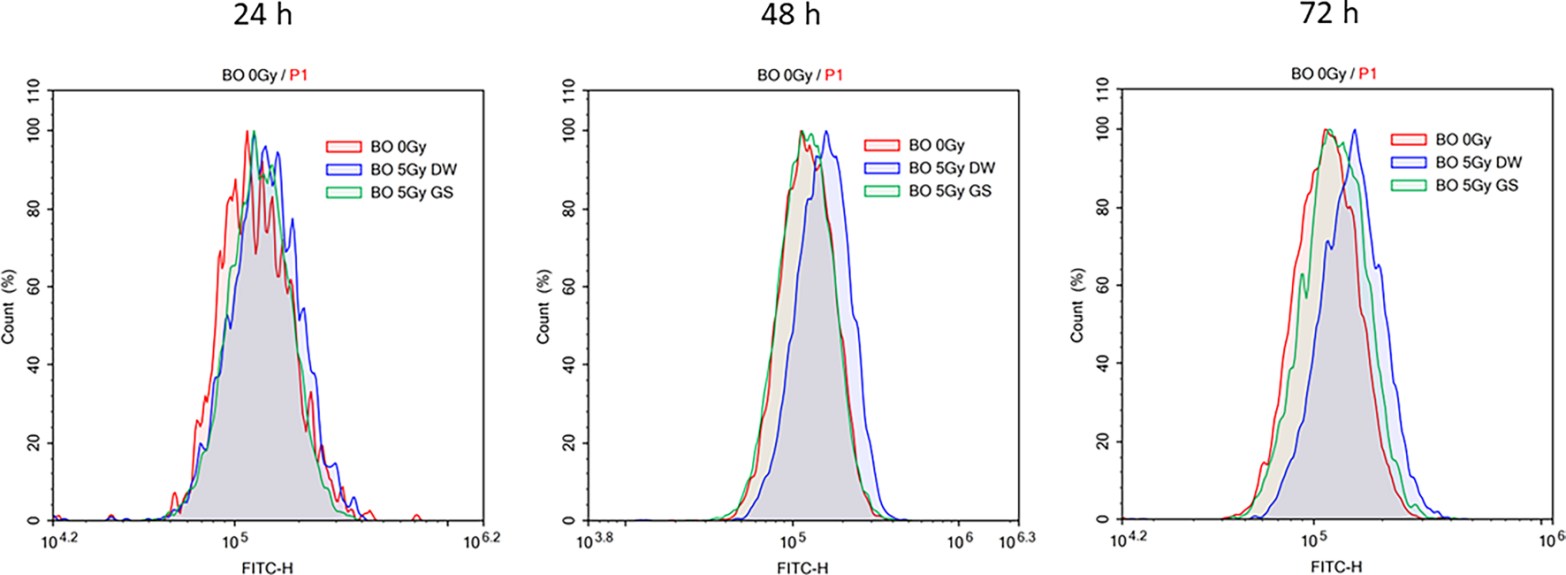
Assessment of cellular lipid peroxidation. After X-irradiation at 5 Gy, cellular lipid peroxidation levels were monitored using a fluorophore, BODIPY™ 581/591 C11. At 24, 48, and 72 h after X-irradiation, the fluorescent intensity of different groups of cells was detected using flow cytometry. Each area represents the levels of non-X-irradiated cells (red), of X-irradiated cells without sericin (blue), and of X-irradiated cells with sericin (green).

GS have various biological properties, including antioxidant and anti-inflammatory properties and is rich in flavonoids. Therefore, we investigated whether the production of reactive oxygen species (ROS) following X-irradiation was reduced by the addition of GS. Our results revealed that intracellular ROS levels at 72 h after X-irradiation was reduced by the addition of GS (Fig. 9). Next, we investigated the level of lipid peroxidation of cellular membranes following X-irradiation. The results showed that the addition of GS also suppressed membrane lipid peroxidation (Fig. 10).

### Cytoprotective effects of various flavonoids and antioxidants in cellular injury caused by X-irradiation

**Figure 11:**
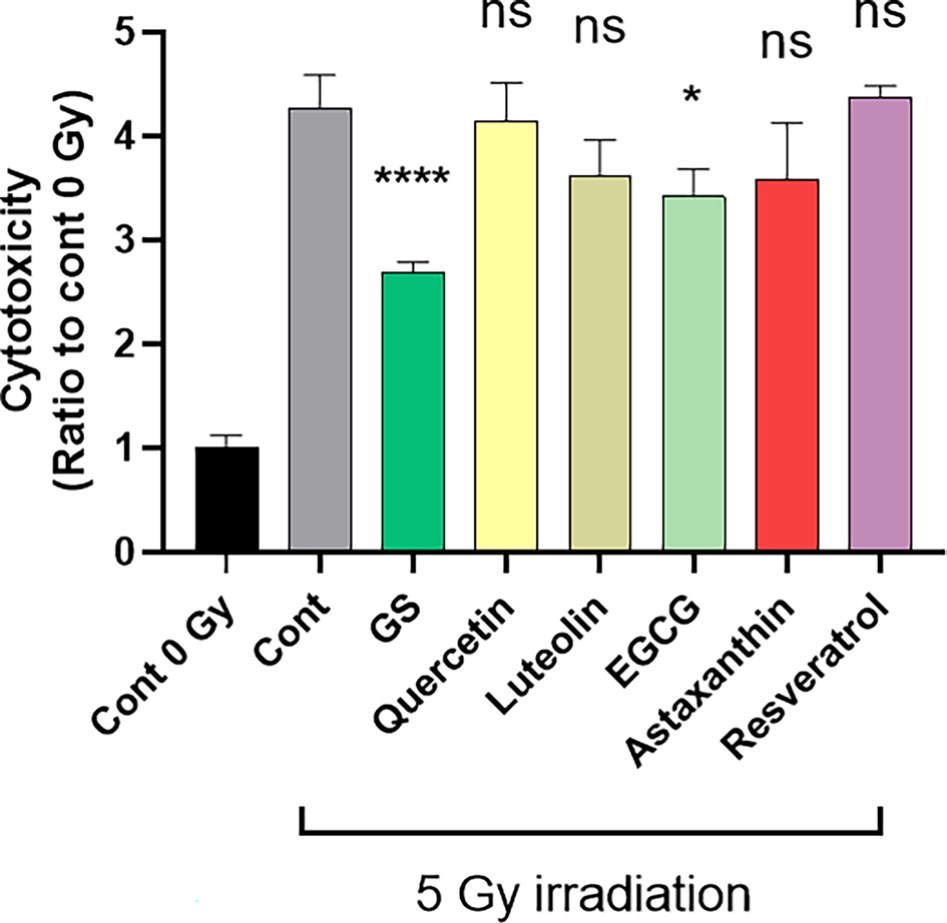
Cell protective effect of various flavonoids and antioxidants in cellular injury by X-irradiation. PSVK1 cells were incubated for 72 h, and then irradiated with 5 Gy. After culturing further 72 h with no additives (Cont), 0.02% sericin (GS), 5 μM quercetin, 5 μM luteolin, 5 μM epigallocatechin-gallate (EGCG), 5 μM astaxanthin, or 5 μM resveratrol, LDH in the medium was measured. Values are the ratio to LDH activity in the medium of the cells without X-irradiation (Cont 0 Gy), and represented the means ± S.D. of at least three determinations. *P* values indicate statistically significant differences with respect to cells in the 5 Gy irradiated control (Cont). *p < 0.05, ****p < 0.001, ns indicates no significance.

GS is known to contain flavonoids such as quercetin. Therefore, we investigated whether several well-known antioxidative compounds alone could reduce cellular damage caused by X-irradiation. Our preliminary experiments confirmed that at 5 µM, the compounds used in the experiment showed no significant difference in LDH values. However, at concentrations above 10 µM, LDH values significantly increased following the addition of these compounds, with or without X-irradiation. Therefore, we decided to use these compounds at a concentration of 5 µM. Quercetin and luteolin are flavonol and flavone, respectively, and at least quercetin is known to be contained in GS as a glycoside. These flavonoids did not show significant cytoprotective effects against X-irradiation (Fig. 11). Astaxanthin and resveratrol, which are also known to have antioxidant capacity, did not show significant cytoprotective effects against X-irradiation. However, a slight cytoprotective effect was observed for epigallocatechin-gallate (EGCG), an antioxidant present in green tea. Although it is not known whether or not GS contains astaxanthin, resveratrol, or EGCG, our observation suggested that the cytotoxicity-reducing effect of sericin on X-irradiation depends at least in part on its antioxidant capacity.

## Discussion

Sericin is a structural protein of silk, consisting of a unique composition of amino acids. When the cocoons of *B. mori* are processed into silk fabric, the sericin is substantially removed and usually discarded in wastewater in the textile industry. However, recent studies have revealed that it is a useful material due to its anti-inflammatory, antibacterial, antioxidant, and photoprotective properties^11^. Kumar et al.^14^ demonstrated that sericin has protective activity against UV radiation-induced skin damage. Radiotherapy is one of the most important cancer treatments. Traditionally, common nonsurgical approach involves radical radiotherapy using conventional fractionation; this is defined as radiotherapy given at a dose of 1.8–2 Gy per fraction, with 1 fraction per day and 5 fractions per week until the prescribed total dose is reached (generally 66–70 Gy for head and neck squamous cell carcinoma). This treatment persists for 6.5–7 weeks^19^. Importantly, one major disadvantage of radiotherapy is the skin damage induced in the irradiated area. Therefore, we decided to investigate whether or not sericin was effective against X-irradiation-induced skin damage. If sericin is shown to be effective against X-irradiation-induced skin damage, it could potentially be used as an injury-reducing material against radiation-induced skin damage in cancer therapy. It is known that green cocoon-derived sericin contains rich flavonoids, and may be useful providers of cytoprotective effects against X-irradiation-induced skin damage. Therefore, in the present study, we investigated the cytoprotective effect of green cocoon sericin against cellular damage induced by X-irradiation using human keratinocytes^20^. LDH is released through the altered cell membrane after the process of cell death. Our study indicated that LDH activity increased over time upon X-irradiation, suggesting that cellular damage caused by X-irradiation increases over time. We observed that the damage caused to keratinocytes by X-irradiation was reduced upon exposure to green cocoon sericin.

The cytotoxic reducing effect of green cocoon sericin was observed even when cells were exposed to high doses of X-irradiation. This cytotoxicity-reducing effect was considered a specific feature of green cocoon sericin, as it was not observed when using white cocoon sericin. Green cocoon sericin is known to contain more flavonoids than white cocoon sericin^18,21^. Reportedly, green cocoons contain quercetin glycoside, kaempferol glycoside, and their aglycons^22^. Some flavonoids have demonstrated antioxidative ability^23^. Inflammation and oxidative stress are generally thought to be associated with radiation-induced skin damage^24^. Therefore, the effect of green cocoon sericin in reducing radiation-induced cellular damage is presumably due to, at least in part, the flavonoids it contains. The amount, type, and form of flavonoids in the green cocoon sericin used in this study are unknown. In the present study, quercetin alone was not found to be effective in reducing cellular injury. Thus, flavonoids glycosides may have a mitigating effect. Notably, although green cocoon sericin dialyzed on a dialysis membrane with a MWCO of 3.5 kDa was significantly less effective in reducing cytotoxicity than green cocoon sericin without dialysis treatment; it was still effective in reducing cytotoxicity when compared with nondialyzed GS. Sericin molecules have a MW > 3.5 kDa^25^. In contrast, flavonoids have a MW < 3.5 kDa. From the results shown in Fig. 7B, the reduction in X-irradiation-induced cell injury was lower in GS dialyzed using the MWCO 3.5 kDa membrane, but still showed a significant reduction in damage. However, GS dialyzed using the MWCO12–16 kDa membrane lost the damage-reducing effect (Fig. 7A). Therefore, we suggest presence of many active ingredients, with some less than 3.5 kDa in molecular weight, while others are between 3.5 and 12–16 kDa. Alternatively, not only flavonoid glycosides but also other components contained in the green cocoon sericin may have been responsible for the reduced cellular damage caused by irradiation. Nonetheless, the green cocoon sericin may show a cell injury-reducing effect by removing harmful molecules, such as ROS produced by X-irradiation^26^. In fact, the addition of GS following X-irradiation was shown to decrease intracellular ROS levels (Fig. 9). GS addition was also shown to suppress membrane lipid peroxidation (Fig. 10). Further analyses are needed to clarify the exact flavonoids and other active ingredients that are responsible for the cytoprotective effect observed in our study. In addition, clarification of the mechanism of action will need to be addressed in future investigations.

In the present study, pretreatment with green cocoon sericin showed no effect on reducing X-irradiation-induced cell injury, whereas adding green cocoon sericin after X-irradiation showed a sericin exposure time-dependent reduction. In addition, delayed application of green cocoon sericin led to reduction in cellular injury. These findings suggest that, for the treatment of radiation-caused skin injury, green cocoon sericin may be applied to the irradiated area directly after X-irradiation or after a day or two. Radiation therapy is generally administered in fractionated low doses. Therefore, applying the green cocoon sericin to the irradiated area at intervals between irradiations may contribute to reducing radiation-induced skin damage. Such care could reduce the extent or delay the onset of skin injury in long-term radiotherapy.

In the present study, we demonstrated that green cocoon sericin exhibits the effect in reducing cellular damage to human skin cells caused by X-irradiation for the first time. Although the effect was examined after a single irradiation in this study, there is room for further investigation to decipher whether this effect can also be demonstrated for multiple low-dose irradiations in clinical radiation therapy. Our study opens new avenue in the treatment of X-ray irradiated skin for patients subjected to radiotherapy.

## Methods

### Materials

Green cocoon sericin (0.78%) and white cocoon-derived sericin (0.59%) were purchased from Kimonobrain (Niigata, Japan). The LDH assay kit (Cytotoxicity LDH Assay Kit-WST) was purchased from Dojindo (Kumamoto, Japan). Unless stated otherwise, all other chemicals and reagents were purchased from Fuji Film-Wako Pure Chemical Industries, Ltd (Osaka, Japan).

### Cell culture

Human keratinocytes (PSVK1) were purchased from the JCRB cell bank of the National Institute of Biomedical Research and Innovation (Japan). PSVK1 cells were cultured in Keratinocyte Growth Medium 3 (PromoCell GmbH, Heidelberg, Germany) at 37 °C, 5% CO_2_, and 95% air. When cells were X-irradiated, the medium was replaced with a medium containing 25 mM HEPES to maintain the pH in the absence of CO_2_, and after X-irradiation, cells were put back into normal culture condition, i.e., in the presence of 5% CO_2_, and the medium was, thus, replaced by a HEPES-free medium.

### X-ray irradiation and measurement of LDH activity

X-irradiation was performed by MBR-1605RA (Hitachi Power Solutions, Ibaraki, Japan) at 160 kV and 5 mA. PSVK1 cells were seeded at 5 × 10^4^ cells in 35 mm dishes, irradiated with 2.5–10 Gy of X-rays 72 h after seeding, and cultured for 24–72 h. The activity of LDH released into the medium by damaged cells was assessed using the LDH assay kit following the manufacturer’s instructions. After the incubation period, 100 μL of culture supernatant was immediately mixed with 100 μL of the kit reagent and incubated in the dark air for 30 min. The absorbance of each sample was then measured at 490 nm. The LDH activity in each treatment group was calculated with the following formula.

LDH activity = O.D. 490 of each sample irradiated/verage of O.D.490 of the control 0 Gy sample.

### Cell viability

Cell viability was determined by trypan blue staining. After collecting the culture supernatant for LDH activity measurement, the cells were washed with phosphate buffered saline (PBS) three times and treated with 0.25% trypsin-EDTA (Thermo Fisher Scientific, Tokyo, Japan). The detached cells were resuspended in 1 mL of fresh medium, and 50 µL of the suspension was mixed with an equal volume of 0.1% trypan blue solution. Trypan blue-negative cells were counted as viable cells using a hemocytometer.

The MTT assay was performed using a CellQuanti-MTT™ Cell Viability Assay Kit (Bioassay Systems, Hayward, CA, USA), according to the manufacturer’s protocol with few modifications. Briefly, 72 h after X-irradiation, cells in 35-mm dishes were rinsed with PBS. Next, 0.4 mL PBS and 75 µL of reagent were added to the cells. After cells were incubated at 37 °C for 1 h, 0.5 mL of solubilizer was added to the cells, and the resulting solution was mixed gently using an orbital shaker for 1 h at room temperature (20 °C). Finally, OD_570_ for each dish was measured.

### Dialysis of green sericin solution

Dialysis of the extract using two different cellulose dialytic membrane tubes was performed to obtain dialyzed green cocoon sericin. First, the sericin solution in Seamless Cellulose Tubing, Small Size 24 with a MWCO of 12–18 kDa (FUJIFILM Wako Pure Chemical Corporation, Osaka, Japan) or a Spectra/Por® 3 with an MWCO of 3.5 kDa (REPLIGEN, Waltham, MA, USA) was dialyzed overnight in 0.9% (w/v) NaCl solution at room temperature. The solution inside the tube was then sterilized using a 0.2-µm hydrophilic cellulose acetate membrane (Sartorius, Goettingen, Germany).

### SDS-polyacrylamide gel electrophoresis (SDS-PAGE)

For SDS-PAGE, each sample was first mixed with an equal volume of a protein denaturing solution (100 mM Tris–HCl (pH 6.8), 20% glycerol, 2% SDS, 0.86 M 2-mercuptoethanol, and 0.2 mM phenylmethylsulfonyl fluoride). SDS-PAGE was performed using a P-PAGEL HR instrument (HER T12.5L, Atto, Tokyo, Japan). After electrophoresis, the gel was stained with 0.25% Coomassie Brilliant Blue.

### Assessment of intracellular reactive oxygen species and lipid peroxidation levels

Intracellular ROS levels were determined using an ROS Assay Kit-Highly Sensitive DCFH-DA (Dojindo, Kumamoto, Japan). This kit is based on a cell membrane-permeable radical probe, 2',7'-dichlorofluorescein diacetate; all procedures were performed according to the manufacturer’s protocol. Briefly, 72 h after X-irradiation, cells were incubated with a fluorescent dye for 30 min. The cells were then detached via a 0.25% trypsin treatment before being resuspended in a serum-free medium. After washing cells twice with PBS, they were resuspended in 0.5 mL of PBS and the fluorescent signal was measured using a flow cytometry system, NovoCyte (Agilent Technologies, Santa Clara, CA, USA) using a 530/30 bandpass filter. In total, 12,000 cells were analyzed using NovoExpress Software version 1.5.0.

Cellular lipid peroxidation levels were monitored using a fluorophore, BODIPY™ 581/591 C11 (Thermo Fisher Scientific, MA, U. S. A.). At 24, 48, and 72 h after X-irradiation, cells were incubated for 15 min with a serum-free medium containing 1 µM of BODIPY solution dissolved in DMSO. Next, the fluorescent intensity of the cells was detected via flow cytometry using the same procedure described above.

### Statistical analyses

All data were analyzed using GraphPad Prism 9.4.1 (Dotmatics, Boston, MA, USA). For paired comparisons, two-way ANOVA followed by Tukey’s multiple comparison tests (Fig. 1) or one-way ANOVA followed by Tukey’s multiple comparison tests (Figs. 2, 3, 4, 5, 6, 7 and 11) was performed.

## Data Availability

All datasets used and/or analyzed during the current study available from the corresponding author on reasonable request.
